# The development and validation of the Virtual Tissue Matrix, a software application that facilitates the review of tissue microarrays on line

**DOI:** 10.1186/1471-2105-7-256

**Published:** 2006-05-17

**Authors:** Catherine M Conway, Deirdre O'Shea, Sallyann O'Brien, Darragh K Lawler, Graham D Dodrill, Anthony O'Grady, Helen Barrett, Christian Gulmann, Lorraine O'Driscoll, William M Gallagher, Elaine W Kay, Daniel G O'Shea

**Affiliations:** 1 Medical Informatics Group, School of Biotechnology, Dublin City University, Dublin, Ireland and National Institute for Cellular Biotechnology, Dublin City University, Dublin, Ireland; 2 Department of Histopathology, Beaumont Hospital and Royal College of Surgeons, Dublin, Ireland; 3 Centre for Molecular Medicine, Conway Institute of Biomolecular and Biomedical Research University College Dublin, Ireland

## Abstract

**Background:**

The Tissue Microarray (TMA) facilitates high-throughput analysis of hundreds of tissue specimens simultaneously. However, bottlenecks in the storage and manipulation of the data generated from TMA reviews have become apparent. A number of software applications have been developed to assist in image and data management; however no solution currently facilitates the easy online review, scoring and subsequent storage of images and data associated with TMA experimentation.

**Results:**

This paper describes the design, development and validation of the Virtual Tissue Matrix (VTM). Through an intuitive HTML driven user interface, the VTM provides digital/virtual slide based images of each TMA core and a means to record observations on each TMA spot. Data generated from a TMA review is stored in an associated relational database, which facilitates the use of flexible scoring forms. The system allows multiple users to record their interpretation of each TMA spot for any parameters assessed. Images generated for the VTM were captured using a standard background lighting intensity and corrective algorithms were applied to each image to eliminate any background lighting hue inconsistencies or vignetting.

Validation of the VTM involved examination of inter-and intra-observer variability between microscope and digital TMA reviews. Six bladder TMAs were immunohistochemically stained for E-Cadherin, β-Catenin and PhosphoMet and were assessed by two reviewers for the amount of core and tumour present, the amount and intensity of membrane, cytoplasmic and nuclear staining.

**Conclusion:**

Results show that digital VTM images are representative of the original tissue viewed with a microscope. There were equivalent levels of inter-and intra-observer agreement for five out of the eight parameters assessed. Results also suggest that digital reviews may correct potential problems experienced when reviewing TMAs using a microscope, for example, removal of background lighting variance and tint, and potential disorientation of the reviewer, which may have resulted in the discrepancies evident in the remaining three parameters.

## Background

Tissue Microarrays (TMAs) provide high-throughput analysis of tissue samples for in situ hybridisation and immunohistochemistry, by means of arranging multiple tissue samples in a uniform structure on the surface of a glass slide. TMAs allow for large numbers of tissue samples to be analysed simultaneously at DNA, RNA or protein level.

Kononen *et al*. first illustrated the use of TMAs in 1998 [1]. The technique involves the excision of cores of varying diameter (0.6 mm to 2 mm) from regions of histological importance on donor tissue blocks and the subsequent insertion of these excised cores into precise co-ordinates on a recipient block. This process is repeated until a two-dimensional matrix of cores is inserted into the recipient block. Once the block is complete, sections can be cut from the block, which are then available for any analysis currently performed on full-face tissue sections.

A large amount of data is associated with TMAs, ranging from information on the tissue (patient information), to their construction, subsequent staining and assessment. It was becoming apparent that applications to assist in pathologist's reviews of TMAs are required, as bottlenecks in the storage and manipulation of the data generated are beginning to emerge.

There have been previous attempts to create software applications that facilitate review of TMAs [2]. The technology has varied from using Microsoft Excel™ spreadsheets, to the creation of complex databases. Manley *et al*. (2001) developed a relational database to store data and images, which focus on clinical outcome [3]. This system consisted of several databases to store TMA images, TMA information, pathological and clinical information, in Microsoft Access™. All data was manually entered into a main online form and then transferred into the relevant database table. Each image was scanned using a grid structure that overlaid the image of the array. The images were composed of six separate 10 × fields, stitched together to form a single image, which was saved as a JPEG image (200–300 kb). However, despite the advances made by this system, rapid file sharing over the internet was limited by large image sizes with slow internet connections. Also, as the software utilised were commercial applications, adaptations to the functionality of the programs were not possible.

Liu *et al*. (2002) utilised a combination of commercial and in-house applications to store data, digitise images and perform statistical analysis [4]. Information was stored in Microsoft Excel™ spreadsheets and reformatted by a program called TMA deconvoluter, into a structure that can be further manipulated to allow statistical analysis and hierarchical clustering. Although Microsoft Excel™ spreadsheets are traditionally used by scientists to store data, there is always a significant risk of human error, as large amounts of data entry are required and the object-oriented nature of the data does not lead to optimal data storage in spreadsheets.

The ability to interpret, review and grade histology in TMA images across the Internet was assessed by Bova et al. [5]. This study evaluated the reviewer's ability to interpret images of TMA cores, in order to assess the presence or absence of prostate cancer and to Gleason grade tumours. In 99% of cases, the images were deemed interpretable; this was done by visual inspection. However, it was suggested that on occasion lengthy downloading times would limit the system's practical use. The authors recommended that compressed files of less than 200 kb should be evaluated for viable image quality, as using files of this size would reduce downloading times. Inter-and intra-observer variability was found to be no greater, and in some cases less than, that reported when using traditional microscope-based Gleason grading. This was evaluated by comparing the Inter-and intra-observer variability observed in Bova et al. study of on line analysis with those previously reported in literature for Inter-and intra-observer of TMA glass slide analysis. When evaluating levels of inter-and intra-observer agreement, Bova et al. used percentage of complete agreement, and k coefficient of agreement. They concluded that web-based technology was an acceptable means to review TMAs. The authors believed a limitation of their study was that web-based analysis was not directly compared with their microscope-based review. The authors recommended that this technology be tested using data resulting from immunohistochemical and in situ hybridisation reviews.

The advent of virtual slides permitted the review of whole tissue slides across the Internet [6]. Virtual slides provide users all the functionality of a microscope, but with numerous additional benefits, including concurrent access for multiple users, tracking of review movements and image annotation. Virtual slides are reminiscent of microscope use, they are favoured by pathologists over static digital images, due to the ability to change magnification and scroll laterally while reviewing the image.

The aim of this study was to develop and validate a software application that would combine the benefits of virtual slides and online relational database technology, to facilitate TMA reviews and scoring via the Internet. To validate the software system developed, a study was created to ensure the system could achieve comparable results to those obtained from traditional glass slide analysis. The study examined users' ability to agree when performing virtual and glass reviews of six immunohistochemically stained tissue microarrays, across eight parameters.

## Results

Two reviewers (Users A and B) examined 183 TMA spots (196 minus 12 control spots and one un-reviewed spot) stained with 3 immunostains using both review methods (microscope and VTM). Neither user reported any technical difficulties when performing digital or microscope analysis. The levels of intra-and inter-observer variability between digital and microscope TMA reviews were assessed, for parameters examining the amount of core and tumour present, the amount and intensity of membrane, cytoplasmic and nuclear staining.

Table 1 illustrates the intra-observer variability between virtual and glass TMA reviews. Good levels of agreement (>60%) between methods were observed when quantifying the amount of core present, the amount of membrane, nuclear and cytoplasmic staining and nuclear intensity. Low levels of agreement between methods were observed when quantifying the amount of tumour present and membrane intensity.

Table 1 illustrates the inter-observer agreement achieved when performing both virtual and glass TMA reviews. Inter-observer agreements achieved when performing virtual TMA reviews were comparable with inter-observer agreements achieved when performing glass TMA reviews, for four out of eight parameters. The Virtual TMA review of the amount of tumour present achieved greater levels of inter-observer agreement than the glass TMA review, of this parameter, however the level of agreement between users for this parameter was very low for both methods of assessment. The glass TMA review of the amount of cytoplasmic staining and intensity, and membrane staining intensity achieved greater levels of inter-observer agreement than the virtual TMA review, of these parameters.

The parameter, % Tumour present, was difficult to assess, with low agreement recorded for inter-and intra-observer agreements across virtual and glass TMA reviews. As this parameter has equivalent levels of inter-observer variability for both virtual and glass TMA reviews, it has to be assumed that poor performance is not based on the method, but more likely the size of the range used. A 10% interval was used to quantify the amount of tumour present, which appears too constrained for this parameter.

**Figure 1:**
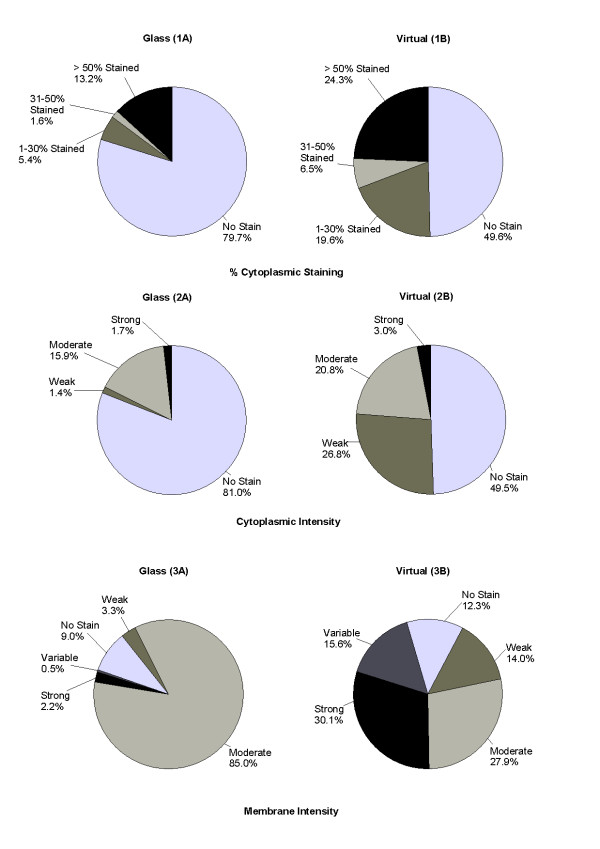
**Distribution of the results for virtual and glass TMA reviews of cytoplasmic and membrane staining intensity and cytoplasmic staining**. Illustrates the distribution of the classifiers when using virtual and glass methods to (1A) Amount of cytoplasmic staining performed by glass TMA review (1B) Amount of cytoplasmic staining performed by virtual TMA review (2A) Cytoplasmic staining intensity performed by glass TMA review (2B) Cytoplasmic staining intensity performed by virtual TMA review (3A) Membrane staining intensity performed by glass TMA review (3B) Membrane staining intensity performed by virtual TMA review.

Reviewers found quantification of the parameter membrane intensity difficult to reproduce between virtual and glass TMA reviews. When users assessed membrane intensity using a microscope, they predominantly used two categories of staining intensity, negative and moderate. With the virtual TMA review, users appeared to use the classifier more extensively, as illustrated in Figure 1. The assessment of intensity of immunohistochemistry, particularly the intensity of membrane bound immunostains is inherently difficult. For example, problems with Her-2 assessment by immunohistochemistry are well documented [7,8].

## Discussion

The objective of this study was to design and develop an online software application that presents tissue microarray images and stores associated review and clinical data. The result was the Virtual Tissue Matrix (VTM), which consists of TMA images available at multiple magnifications, scoring forms to gather TMA review data and a relational database to store the generated results.

The VTM displays virtual TMA images via a web site and facilitates the storage of TMA data via a relational database. There are numerous advantages of using the VTM over other proposed software systems of its type. Downloading of the images is rapid. Only views that are requested by the user are returned at maximum resolution, thereby downloading the minimum required dataset. The VTM was designed in consultation with scientists and pathologists and, as a result, the reviewing process emulates the workflow involved in conventional TMA reviews. The VTM interface is delivered in HTML, via a conventional web browser, allowing for intuitive user interaction. The VTM database is relational; a structure more suited to the storage of the object oriented dataset generated from TMA experimentation, than previous efforts incorporating flat files and spreadsheets for data storage.

Since the creation of the VTM there have been numerous advances in the technologies used for image acquisition [9-11] and image analysis techniques and applications have been well documented in literature [12-17]. Integrated intuitive systems are now available that rely on minimal human intervention when scanning slides such as Aperio or Dmetrix [18]. Numerous commercial image acquisition applications are now available[19-21]; however, cost of purchase is often high for these integrated systems putting them out of reach for many research laboratories.

The VTM has been upgraded to support images generated by an Aperio Scanscope T3 Scanner™. Advantages of using the Aperio Scanscope T3 Scanner™ include, batch uploading of slides, the ability to scan glass TMA slides at 20 × magnification within minutes, one touch scanning which reduces manual intervention, automatic section of autofocus points within the tissue, and seamless images with no tiling artifacts. Despite the advances in image acquisition and the obvious advantages automated systems have over older more labour intensive systems, these systems do not wholly address the problem of relating TMA images to review and image analysis data.

The method used to acquire digital images within the VTM is not the main concern of this manuscript, the technology is constantly developing and advancing, and as new and improved systems are developed they can be integrated into the VTM with ease as illustrated by the upgrade to Aperio™.

Once developed the VTM was validated, via assessment of inter-and intra-observer variability on two users' evaluations of immunohistochemically stained tissue microarrays, using digital and microscope analysis. Eight parameters were evaluated, the amount of core and tumour present, the amount and intensity of membrane, cytoplasmic and nuclear staining.

Comparisons evaluated in this study illustrated that intra-and inter-observer virtual TMA reviews produced equivalent levels of agreement as intra-and inter-observer glass TMA reviews, for five out of the eight parameters examined. Where discrepancies occurred it was dependent on the parameters and users involved.  In all comparisons, low levels of agreement for the amount of tumour present were observed. This was not surprising, as the application of classifiers to any data continuum (data that does not naturally fall into discrete clusters) results in scoring variability around the interfaces of the classifier. This variability is increased when the number of classes are increased creating more interfaces. Also, of the two reviewers used, one was a scientist and one a pathologist. The scientist accurate interpretation of tumour/non tumour may potentially be questioned as a result of this work.

Of particular interest, were a large number of observations that were considered positively stained by virtual TMA reviews which were considered negatively stained when reviewed using a microscope. This was particularly evident when quantifying the amount of cytoplasmic staining; where virtual TMA reviews observed substantially more positively stained spots than glass TMA reviews. The additional positively stained spots were largely considered to stain between 1–30% of the tumour area and/or to be weakly stained. This suggests that virtual TMA reviews may be more successful in allowing the identification of small areas of staining and/or where staining intensity is low.

One proposed reason for the identification of staining when using digital images that was not observed with a microscope was the use of correcting adjustments to the image data during the digitising of TMAs. Bulbs used in microscopes have a characteristic tint; in general this is yellow or straw coloured. However, this tint is removed when digitising slides using a corrective algorithm, potentially unmasking weak staining that would otherwise be attributable to background tint. Also, with microscope based analysis, background light is adjusted to best suit each individual spot. When digitising the slides for this study, a constant background light intensity was used to digitise all slides for this study.

Excluding nuclear staining, where positive staining was infrequent, agreement levels were low when examining staining intensity. When using a conventional microscope, in general, users failed to utilise all grades within the classifier to characterise positive staining intensity; the category of moderate staining was repeatedly used when positive staining was observed, particularly for membrane staining. However, with digital reviews, all grades within the classifier were utilised more extensively, which suggests that the review of digital images gives a user more confidence to discriminate between different intensities and that subtle differences in intensity may be easier to detect when utilising digital slides, than when utilising a microscope. This may be due to the standardisation in lighting while preparing the images.

Human observers, while excellent at object classification, are inherently poor at quantifying intensities and areas to any degree of accuracy. Studies have shown that image analysis produces more reproducible results than pathologists for quantifying the intensity of staining, in relation to β-Catenin expression in TMAs for colon cancer [22]. Image analysis systems may identify subtle differences in staining intensity, which are not quantifiable by a human reviewer, thus leading to the better correlation of expression data to prognostic indicators.

## Conclusion

The virtual tissue matrix (VTM) was created to assist in TMA analysis, by providing digital TMA images at multiple magnifications online, and submitting TMA review data from an online form into an associated database. The VTM illustrated that digital TMA analysis obtained equivalent levels of agreement as microscope based analysis, for five out of eight parameters. The remaining three parameters achieved greater levels of agreement when performed using microscope analysis. However, on further investigation of the three parameters, it is proposed that the digital reviews may be providing the user with greater capability to accurately assess staining presence and intensity. Results illustrated users were incapable of agreeing when comparing digital and microscope TMA analysis when classifying staining intensity. Greater levels of staining was observed when performing digital TMA analysis, it is suggested this is due to the background correction step involved in digitising the slides.

Comparisons of digital with glass reviews of immunohistochemistry stained slides is well documented in literature, however, in order to validate the VTM it was necessary to perform this study. As previously reported in literature, there was some degree of inter-and intra-observer variability. However, the ability of users to observe more positive staining when performing digital reviews, and the inability of users to utilise all categories within the classifiers provided when performing glass reviews are previously unreported in literature.

## Methods

### Construction of TMAs

Forty eight bladder tumours which were part of a wider study were utilized in this evaluation. Bladder cancer TMAs were constructed from formalin-fixed paraffin-embedded (FFPE) tissue blocks using a Beecher Instruments^® ^tissue arrayer. All FFPE blocks were sectioned and stained with Haematoxylin and Eosin (H&E). Two cores each of normal and tumour tissue where sampled. A total of 6 TMAs were constructed, with 48 cases and 196 2 mm cores, including 12 control spots composed of liver tissue. Due to the heterogeneous nature of bladder tissue, 2 mm cores were utilised. TMAs were sectioned at 4 μm and probed with three antibodies, E-cadherin (Novocastra™), β-Catenin (Labvision Corp., RB-9035-P1) and PhosphoMet Tyr1234/1235 (Cell Signalling Technology, 3126S). The method used was the Vectastain^® ^ABC (avidin/biotin) system (Vector Laboratories Inc, PK6200), with visualisation being accomplished by using DAB (3,3'-Diaminobenzidine tetrahydrochloride) as the chromogen. The antibody to E-Cadherin shows membrane staining; the antibody to β-Catenin shows membrane and cytoplasmic staining [23], the antibody to Phospho met shows membrane staining, cytoplasmic staining and nuclear staining [24].

### Construction of virtual TMAs

The imaging system was composed of an Olympus BX-40 microscope (Olympus, NY, USA) incorporating a Prior H101 motorised stage. Images were captured at 4 × using a Plan Achromat lens, and at 20 × using a Plan Fluorite lens. The camera used to scan the immunostained TMAs was a 3-chip JVC KY 55 B 3 CCD. The camera has a red, green and blue (RGB) digital signal output to an Imaging Technologies IC RGB frame grabber, (Coreco Imaging Incorporated, MA, USA) which was housed in a Silicon Graphics ZX10 imaging workstation. A software algorithm was constructed using the Optimas development environment (Media Cybernetics, MD, USA) that facilitated the remote control of the stage and the construction of wide field-of-view images from a montage of smaller fields. All software was written in-house. This facilitated the development of a low cost method of image acquisition. Purchase of dedicated scanners for image acquisition can often be prohibitively costly for research groups, and this approach may present researchers with a low cost solution to this issue.

TMAs were initially scanned at 4 × to create a tiled 'thumbnail' image of the entire array. This overview image was used to locate cores manually through a custom Graphical User Interface. The user clicked on the centre of each core on the overview image and the coordinate generated was used to seed an automatic scanning algorithm for all cores at 20 ×. The array of captured images (6 × 8) were then tiled together to form a montage bitmap image of approximately 60.5 MB (4607 × 4592 pixels. 2.0 mm cores were used as they illustrate the ability of the scanning system to acquire and integrate multiple fields of view at 20 ×, a large diameter of core was required as a smaller diameter cores could potentially be captured within a single field.

The Macromedia Flash application, Zoomify™, was used to display images within the VTM framework. Zoomify™ Droplet, a software tool provided by Zoomify™, uses the original scanned BMP image as an input and converts it into a set of JPEG image tiles [25]. This tileset, once uploaded to a webserver, can be displayed via the Internet using the Zoomify™-embedded object within a conventional web page. Zoomify™ initially presents the user with a low power view of each TMA spot. The users can then scroll around the image and when required zoom into a maximum magnification of 20×.

### Validation of image quality

**Figure 2:**
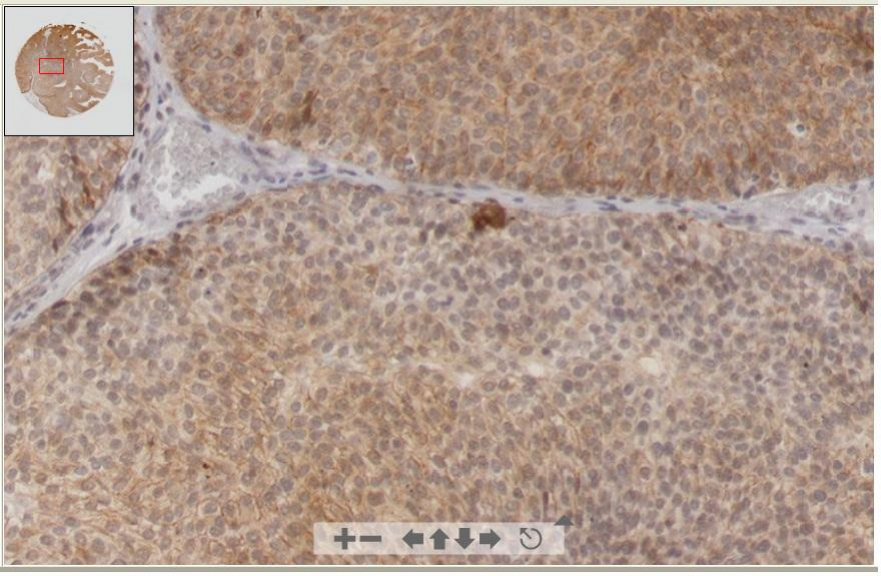
**Digital image of TMA spot presented in VTM using Zoomify™ application**. On the top left corner of image is the thumbnail overview; the red box identifies location within the spot. The key at the bottom of the image allows the user to change position or magnification, which can also be controlled by the cursor.

When using a lossy compression algorithm, such as JPEG, image quality is reduced when compared with that of uncompressed images. To ensure that the compression rate used by Zoomify™ provided images of sufficient quality, a series of consultations with pathologists and scientists were performed, where compressed and uncompressed images were compared. The outcome of this consultation was that the images generated by Zoomify™ were suitable for scoring. Figure 2 illustrates the quality of images available in the VTM.

### Design phase of the VTM

The design objectives of the VTM system were to provide TMA images of sufficient quality to review over the World Wide Web, to present scoring forms to record TMA results and to create a relational database that can store and subsequently retrieve data gathered during scoring.

PHP, Javascript, HTML and Oracle were used to create the VTM. PHP is a server side scripting language, which creates dynamic web pages, through embedding PHP code in HTML pages. Through the use of SQL queries, it can also extract data from many conventional databases (Oracle, MySQL, etc). Javascript adds interactive client side functionality to otherwise static HTML pages [26]. An Oracle relational database was used to store all the information generated in this study [27].

### Database design

The construction of TMAs facilitates the generation of hundreds of TMA slides from a single TMA block; therefore every TMA slide produced has the ability to be stained with a unique immunostain. A major benefit of storing TMA images and results within a relational database is the ability to extract all the results associated with a single core, which potently may have been immunostained hundreds of times. This functionality is available within the VTM.

Based on analysis of conventional TMA datasets, it was established that eleven groupings of data would be sufficient to record all relevant information; therefore, eleven tables where created in the VTM relational database. Each table contains information relating to a specific aspect of a TMA review. For example, the USER table contains information relating to users only; SCORES table contains information relating to the TMA analysis results only. A unique identifier interlinks all tables and by using SQL statements, information can be retrieved from multiple tables simultaneously. For example, results relating to a specific user can be obtained by creating an SQL statement that requests information from the SCORES and USER tables. Table 2 lists all the tables that exist in the database and examples of the information they record. A complete schema of the database is available [28] and also as Additional file 1.

The database has been designed to eliminate replication of data input. For example, a manufacturer of TMAs details are only entered into the database once regardless of the number of TMAs they have constructed, once their account is created they can be associated with multiple blocks/slides and studies. There is also the ability to add information into the database after the initial study has been created. For example, if patient or biopsy information is not known at the time of entering the TMA review results into the database, they may be entered at a later date, by simply entering the patient or biopsy information and then selecting the cores the information applies to. However, users are unable to overwrite the TMA review results already present in the database.

Depending on the internet connection used (times described here are for LAN settings of 10 Mbps), it takes approx 5 seconds to download the thumbnail overview of a TMA slide; once a core has been selected it takes approx 2–3 seconds to view the individual core in the Zoomify ™ window. There are no restrictions on the number of images that can be displayed within the VTM, currently there are 196 images. However, the number of images displayed is dependent on the capacity of the server available to the users.

### Interface design

The user interface had to be easily navigated, interpretable and provide images at sufficient speed and resolution for review. There are two types of user account within the VTM, namely administrator or user. A user has restricted access to the site; they can only review and score images. An administrator has additional privileges within the site; they can create new studies, add new images and scoring forms and can view all data stored in the database.

**Figure 3:**
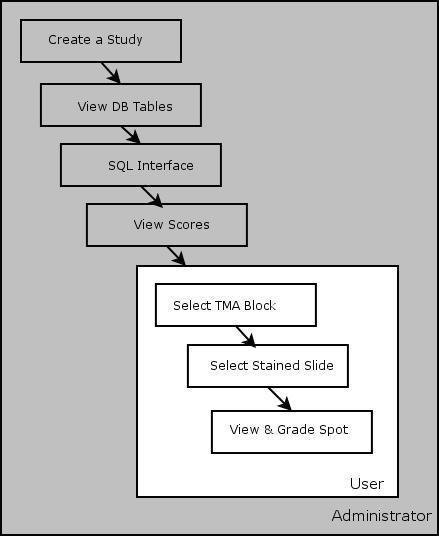
**Options available to users through the VTM interface**. Schematic diagram showing options available to Administrators and Users within the VTM interface.

**Figure 4:**
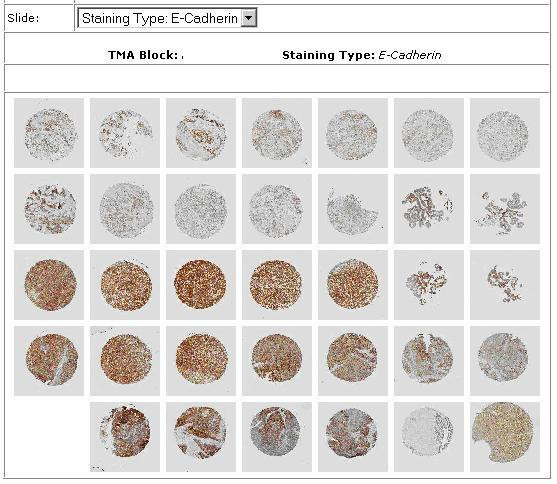
**Overview of digital TMA slide as presented in the VTM interface**. Clicking on any spot will result in an enlarged version of the spot being provided, as in Figure 5.

**Figure 5:**
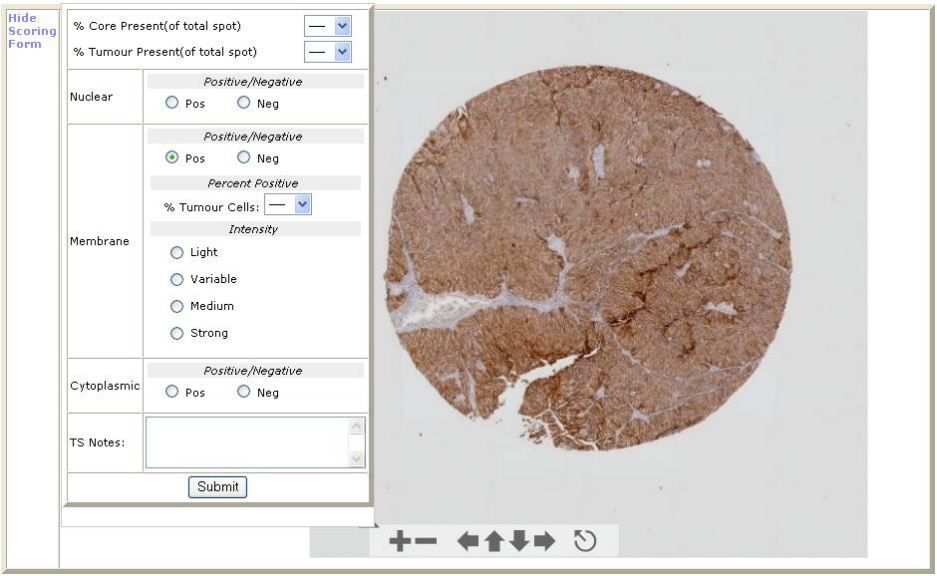
**Scoring form presented to users within the VTM interface**. Results can be entered into the scoring form on the left, the image can be magnified and scrolling is possible via the controls provided.

The VTM was constructed so the user is lead through the site, not having to concentrate on the sequence of events, freeing up analysis time for the reviewing process. Figure 3 illustrates the options available to both user and administrator within the VTM. Figure 4 illustrates the user's view of a TMA slide before a specific spot is selected. A spot must be clicked on to view a scaleable image. Once selected, the spot is reviewed in a pop-up window and the option to record results via a scoring form is presented. Figure 5 illustrates the spot with the associated scoring form. The administrator has the ability to create new scoring forms depending on the user's specifications.

### Users, scales and parameters recorded

Two pathologists and one research scientist scored the immunohistochemically stained TMA slides. One of the former was an external examiner, completing a virtual review of one slide from an international location. This review was conducted to ensure the VTM functioned in a remote location and to ensure that a user who had no contribution to the design phase could use the VTM software system. As a result of having only one data set from this user, their observations were not included in the results that follow.

Review methods included a glass slide review and a virtual slide review. The glass slide review was a traditional microscope-based process; the virtual review was performed over the World Wide Web using the VTM. Eight parameters were examined for each spot. The amount and intensity of membrane, cytoplasmic and nuclear staining, as well as the amount of core and tumour present in each spot, were recorded. A five-point scale was used to record the staining intensity. Within the five-point scale, grade 0 represented negative staining, grade 1 was weak staining, grade 2 was moderate staining and grade 3 was strong staining. Grade 4 was included to record variable staining; this option was only available when scoring membrane staining.

A four-point scale was used to record the amount of staining present in tumour-containing regions of each spot. Grade 0 represented no staining, grade 1 represented 1–30% of relevant cells staining, grade 2 represented 31–50% staining and grade 3 represented greater than 50% staining. An eleven-point scale was used to record the amount of core and tumour present. Grade 0 was negative/no tissue, and grade 1 to 10 increased in 10% increments to 100%.

### Cohen's un-weighted Kappa

Cohen's un-weighted kappa values were one of two methods used to quantify the level of agreements achieved when comparing two datasets generated from TMA reviews. Kappa was not the primary statistics used in the dataset comparisons involved in this study, however; Kappa has been included as it is widely used in comparisons of observer agreement of this type. Landis and Koch kappa interpretation scale was used to evaluate the level of kappa agreements; the interpretation scale is illustrated in Table 3[29]. The predominant method used to quantify the levels of agreement achieved when comparing two datasets generated from TMA reviews, was the percentage of cases where the two datasets were in complete agreement.

## Abbreviations

TMA-tissue microarray

TMAs-tissue microarrays

VTM-virtual tissue matrix

## Authors' contributions

CMC involved in designing the VTM, performed validation of the VTM and TMA virtual image quality, performed statistical analysis, and drafted the manuscript. DOS involved in designing the VTM, validation of TMA virtual image quality, constructed TMAs, performed immunohistochemical staining and performed microscope and digital TMA reviews. SOB involved in designing the VTM, and proof reading the manuscript. DKL designed the VTM database, digitised the slides, executed the coding of the VTM and performed validation of the VTM. GDD created the first version of the VTM, involved in designing the current VTM. AOG involved in designing the VTM, and validation of TMA virtual image quality. HB performed microscope and digital TMA reviews. CG performed digital TMA reviews. LOD proof read the manuscript. WMG involved in designing the VTM, validation of the TMA virtual image quality, structuring and proof reading the manuscript. EWK involved in designing the VTM, validation of the TMA virtual image quality, structuring and proof reading the manuscript. DGOS conceived the VTM application, conceived the validation study (comparing microscope and digital TMA slide reviews), participated in VTM design, creation and validation, coordinated and helped to draft and proof read the manuscript. All authors read and approved the final manuscript.

## Availability and requirements

The Virtual Tissue Matrix can be found at . Operating system(s): Platform independent; Programming language used include, Javascript, PHP, HTML. Other requirements include Macromedia flash version 7. The source code of the VTM is available at Additional file 2.

## Supplementary Material

Additional File 1 A complete schema of the database structure, and lists all the tables and entries within the tables. Click here for file

Additional File 2 Source code for the VTM site and database Click here for file

## Figures and Tables

**Table 1:** Agreement levels (%) and un-weighted kappa values by measured parameter for each comparison of TMA reviews.

**Parameters**	**Virtual vs. Glass**		**User A vs. B Virtual**		**User A vs. B Glass**	
	% Agree	Kappa	% Agree	Kappa	% Agree	Kappa
% Core Present	71.3	*0.507*	67.1	*0.414*	67.4	*0.448*
% Tumour Present	47.3	*0.407*	37.9	*0.283*	33.0	*0.244*
% Membrane Staining	81.2	*0.577*	78.2	*0.553*	78.6	*0.520*
% Cytoplasmic Staining	64.0	*0.37*3	42.8	*0.198*	65.9	*0.280*
% Nuclear Staining	85.0	*0.226*	84.9	*0.524*	95.8	*0.239*
Membrane Intensity	32.4	*0.167*	25.3	*0.153*	77.1	*0.447*
Cytoplasmic Intensity	58.2	*0.306*	50.8	*0.263*	65.2	*0.234*
Nuclear Intensity	82.1	*0.109*	83.3	*0.474*	89.2	*0.234*

Virtual vs. Glass was the average of two users' agreements when comparing virtual with glass TMA reviews. User A vs. B Virtual was User A virtual TMA review compared with User B virtual TMA review. User A vs. B Glass was User A glass TMA review compared with User B glass TMA review.

**Table 2:** Tables within the VTM database and examples of their content.

**Table Name**	**Information held**	**Examples**
User	User details	E-mail address
TMA Manufacturer	Manufacturer details	Name
TMA	Tissue Microarrays	Diagram of cores
Patient	Patient information	Gender
Biopsy	Biopsy details	Biopsy notes
Core	Core details	Tissue type
Spot	Spots in a slide	Magnification scanned
Experiment Slide	Experiment procedure	Treatment name
Slide scoring range	Scoring ranges	Staining category
Score	Results	Amt of Nuclear staining
Intensity Table	Staining intensity details	Staining type

**Table 3:** Interpretation of Landis and Koch kappa values.

**Kappa Value Range**	**Interpretation**
0–0.2	Slight
0.2–0.4	Fair
0.4–0.6	Moderate
0.6–0.8	Substantial
0.8–1.00	Almost Perfect

Ranges of Cohen's Kappa values.
