# Supplementary figures and images for: The development and validation of the Virtual Tissue Matrix, a software application that facilitates the review of tissue microarrays on line
Source: BMC Bioinformatics. 2006 May 17;7:256. doi: 10.1186/1471-2105-7-256 (PMC1479843; doi:10.1186/1471-2105-7-256)

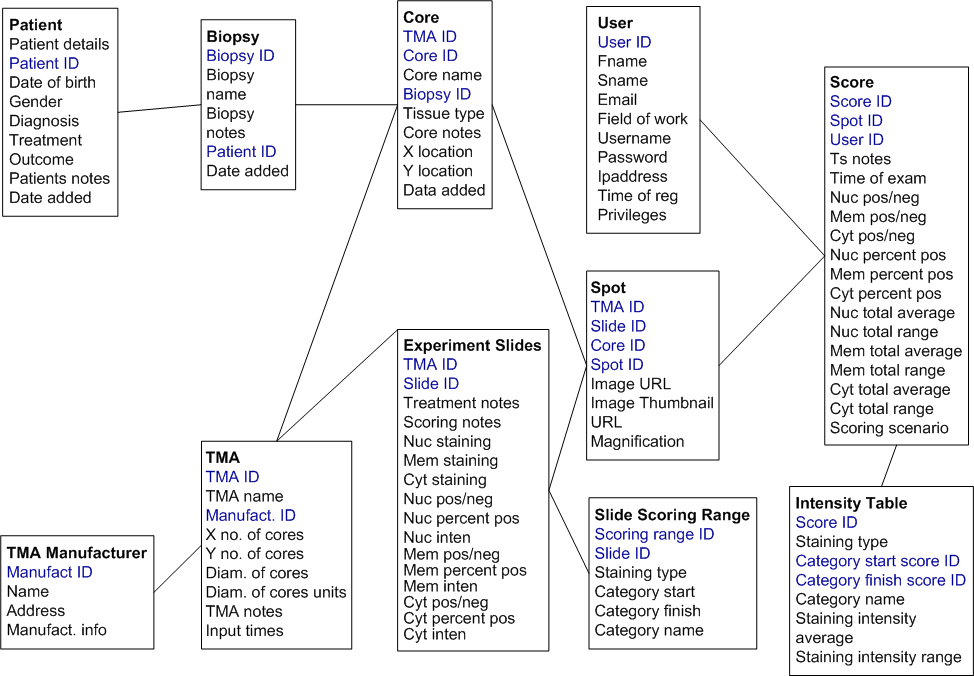

Supplement: Additional File 1 — A complete schema of the database structure, and lists all the tables and entries within the tables. [file 1471-2105-7-256-S1.bmp]

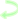

Supplement: Additional File 2 — Source code for the VTM site and database [file 1471-2105-7-256-S2.zip › Source Code vtm/images/arrow.png]

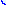

Supplement: Additional File 2 — Source code for the VTM site and database [file 1471-2105-7-256-S2.zip › Source Code vtm/images/bl.bmp]

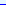

Supplement: Additional File 2 — Source code for the VTM site and database [file 1471-2105-7-256-S2.zip › Source Code vtm/images/bottom.bmp]

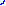

Supplement: Additional File 2 — Source code for the VTM site and database [file 1471-2105-7-256-S2.zip › Source Code vtm/images/br.bmp]

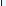

Supplement: Additional File 2 — Source code for the VTM site and database [file 1471-2105-7-256-S2.zip › Source Code vtm/images/left.bmp]

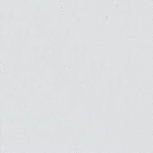

Supplement: Additional File 2 — Source code for the VTM site and database [file 1471-2105-7-256-S2.zip › Source Code vtm/images/non-transfered.jpg]

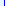

Supplement: Additional File 2 — Source code for the VTM site and database [file 1471-2105-7-256-S2.zip › Source Code vtm/images/right.bmp]

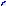

Supplement: Additional File 2 — Source code for the VTM site and database [file 1471-2105-7-256-S2.zip › Source Code vtm/images/tl.bmp]

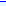

Supplement: Additional File 2 — Source code for the VTM site and database [file 1471-2105-7-256-S2.zip › Source Code vtm/images/top.bmp]

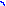

Supplement: Additional File 2 — Source code for the VTM site and database [file 1471-2105-7-256-S2.zip › Source Code vtm/images/tr.bmp]
