# Supplementary material for: The development and validation of the Virtual Tissue Matrix, a software application that facilitates the review of tissue microarrays on line
Source: BMC Bioinformatics. 2006 May 17;7:256. doi: 10.1186/1471-2105-7-256 (PMC1479843; doi:10.1186/1471-2105-7-256)
Supplement: Additional File 2 — Source code for the VTM site and database [file 1471-2105-7-256-S2.zip › Source Code vtm/index.php]

TMA 2


php
$prob = $\_GET['prob'];
if(isset($prob))
{
if(strnatcasecmp ($prob, "noMatch") == 0)
{
print "<i**No matching username and password were found in the database.**

";
}
else if(strnatcasecmp ($prob, "notEntered") == 0)
{
print "***Please enter a username and password.***

";
}
}
?>

***Please insert your Username and Password***
  
  
  

|  |  |
| --- | --- |
| Username: |  |
| Password: |  |
|  | |
| Admin Password: Not Necessary |  |
|  | |

  
  
*Any queries please E-mail: **telepathology@dcu.ie***
