# Supplementary material for: The development and validation of the Virtual Tissue Matrix, a software application that facilitates the review of tissue microarrays on line
Source: BMC Bioinformatics. 2006 May 17;7:256. doi: 10.1186/1471-2105-7-256 (PMC1479843; doi:10.1186/1471-2105-7-256)
Supplement: Additional File 2 — Source code for the VTM site and database [file 1471-2105-7-256-S2.zip › Source Code vtm/How to connect to Oracle DB.doc]

In order to connect the VTM to an Oracle Database:

Open functionfile.php

On line 6 replace 'databaseUsername' with the name of the Oracle database you wish to connect to.

On line 6 replace 'databasePassword' with the password for the Oracle database you wish to connect to.
